# Preparation and characterization of ancient recipe of organic Lime Putty-Evaluation for its suitability in restoration of Padmanabhapuram Palace, India

**DOI:** 10.1038/s41598-021-91680-8

**Published:** 2021-06-24

**Authors:** M. Shivakumar, Thirumalini Selvaraj, Magesh Peter Dhassaih

**Affiliations:** 1 Department of Structural and Geotechnical Engineering, Vellore Institute of Technology, Vellore, 632014 India; 2 Department of Structural and Geotechnical Engineering, Vellore Institute of Technology, Vellore, 632014 India; 3 Ministry of Earth Sciences, National Institute of Ocean Technology, Pallikaranai, Chennai, 600100 India

**Keywords:** Chemistry, Energy science and technology, Engineering, Materials science

## Abstract

The study aims at preparation and characterization of six organic lime putty (hydraulic Lime + fermented plant extract) using regionally available plants namely *Terminalia Chebula (*kadukkai*), Rosa Sinensis* (hibiscus*), Palm jaggery* (refined sugar), *Xanthorrhoeaceae* (aloe vera), and *Indigofera Tinctoria* (neelamari) as per the methods given in the ancient palm leaf of Padmanabhapuram Palace, India. Advanced analytical techniques like Gas chromatography-mass spectroscopy (GC–MS), UV-Spectrophotometer and carbon dioxide quantification were used to study the fermented plant extracts and Fourier transform-infrared spectroscopy (FT-IR), X-ray Diffraction (XRD), Field emission-scanning electron microscopy (FESEM) to study hydrated phases and microstructure of organic lime putty. GC–MS recorded the phytochemical compounds like fatty acids, traces of proteins, polysaccharides and carbohydrates. Fermented kadukkai and neelamari extracts reported as fatty acid, palm jaggery as carbohydrate, hibiscus as polysaccharide and aloevera rich in all the biomolecules. The detection limit of Quantification:0.013 and limit of detection:0.067 for polysaccharides, 0.026 and 0.088 for unsaturated fatty acids was reported through a U.V spectrophotometer for all the herbs. Aloevera and neelamari fermented extracts recorded the CO_2_ release around 96,000 and 90,000 ppm on 4th day of fermentation, whereas for other herbs it ranged below the recorded readings. Supply of CO_2_ has initiated the internal carbonation of the lime putty and precipitation of calcite in three different forms aragonite, calcite and vaterite minerals. The addition of organics resulted in high-intensity portlandite peaks and calcium carbonate polymorphs as reported in XRD graphs in agreement with FT-IR analysis. FESEM morphology validated the early formation of carbonate polymorphs, and EDX. has shown that kadukkai lime putty, jaggery lime putty and reference lime putty. mixes have calcium around 35–45%. From the overall results, 3% addition of eco-friendly biopolymers has altered the properties like setting time, water repellency and higher carbonation rate, which is the main reason behind longevity of the structure.

## Introduction

**Figure 1:**
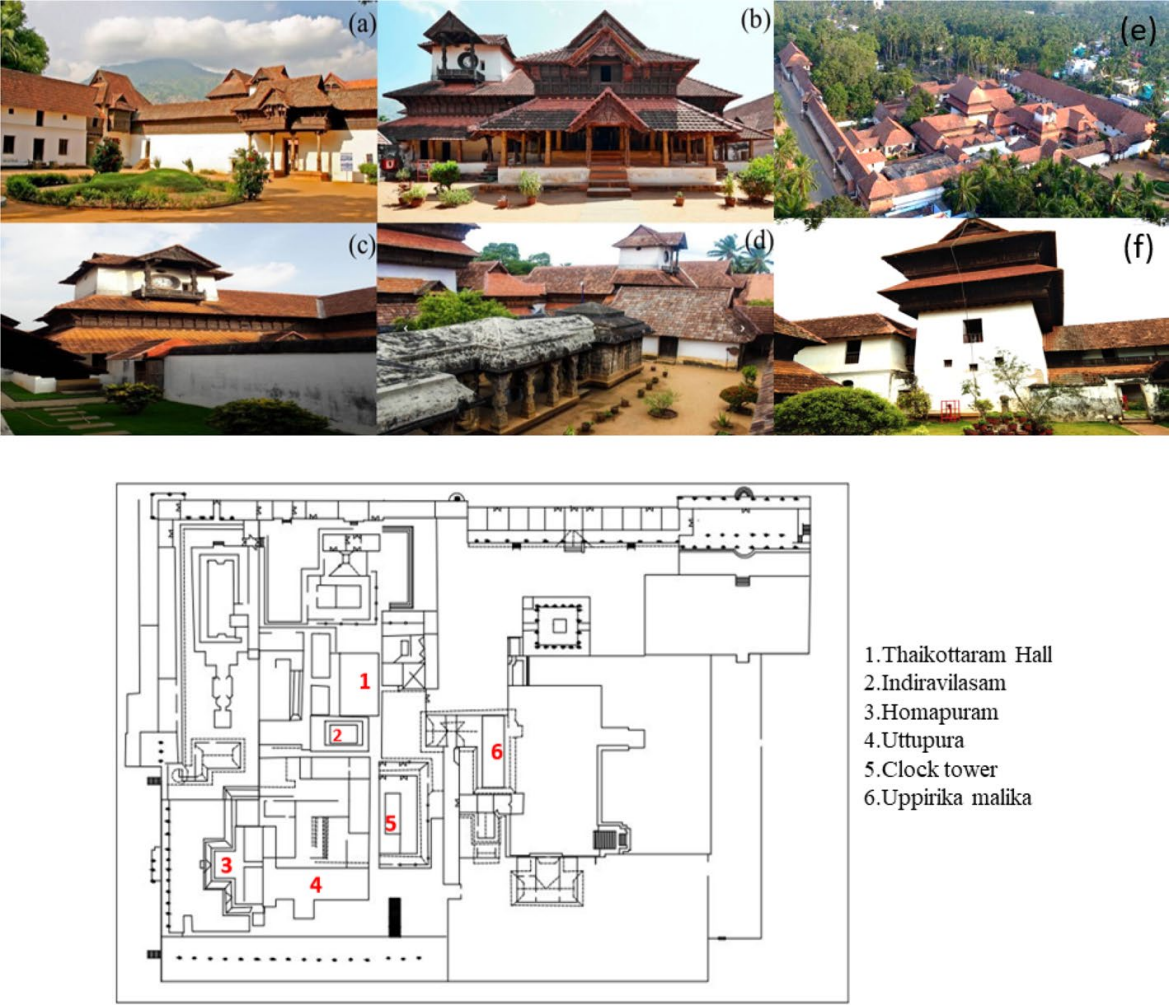
(**a**) Padmanabhapuram Palace plan, (**b**) Poomukha Mallika (Entrance), (**c**) Mani meda (Clock tower), (**d**) Thaikottaram hall, (**e**) Aerial view of Palace, (**f**) Uppirika Malika.

Since prehistoric times, India abides by the vast cultural heritage with the glorious tradition of preserving knowledge through oral and written communication. Historic structures include the temples, palaces, tombs & mosques, and the buildings, artefacts, historic precincts, and cultural landscapes^1–5^. Moreover, the ancient materials and the protected archives like literary collections, metal carvings, palm leaf manuscripts, wall paintings and stone inscriptions were the earliest historic references symbolizing the regional styles, agama shastras (religious rituals) and Chitra sutra (mural paintings) before the invention of the paper^6–9^. The earliest known palm-leaf manuscript belongs to the 2nd Century A.D of the Gupta regime. Writing on palm leaf continued up to 19th Century A.D, archiving the historical significance of civilization. Heritage is any tangible or intangible values passed on to us from the past. This wisdom hauls various traditional significances like economic, cultural, aesthetic, spiritual and archaeological sources that connect with past civilization and carry their advancements to modern times. Likewise, some ancient sources communicate the knowledge, ranging from the caves to copper plates and from the bark of trees to leaves of various kinds^8,10–12^.The ancient manuscripts are the sources of human history available on various media like stone, clay, tablets, palm leaves, metal leaves, barks, animal skin, cloth, paper etc. Despite all the ancient writing materials, palm leaf was used as the predominant media due to the local availability of palm trees across the Indian subcontinent^10^. Though many modern innovations have emerged in recent times, not all are holistic. Few recent studies underway understand the ancient structures' holistic construction practices through digital preservation, artificial intelligence, and modern analytical techniques. Starting from the Indus valley providing the evidence on the ancient town planning of Harrapan and Mohenjo-Daro depict the building culture, advanced drainage system and road systems. The construction method has transformed since 300 B.C with the whole new level of Dravidian style of gigantic towers. Long pillared halls, artistic vimanas and common structural features such as domes, slender minarets and arches were influenced from the Islamic and the Persian style of architectures. Every structure has its unique significances and architectural point of view. One such fine example of Brihadeshwara temple, Tanjore, India has the longest vimana of height 216 ft with axial and symmetrical geometry rules with fortified granite structure in the world^5^. The archaeological archive forum across the countries has documented some of the traditional secrets of old construction practices and preservative methods to understand the designs' scientific methods. It is estimated that there are 300 manuscript libraries all over India and 77 libraries outside India on preserving the archives. Some of the major repositories in India are national archives of India (N.A.I.), Indian national trust for art and cultural heritage (I.N.T.A.C.H.), Indira Gandhi national center for the arts (I.G.N.C.A.), Department of culture and its museum and libraries, state archives and state museums. Mostly the national archives reveal the secrets of regimes, culture, medicinal scripts, trades and civilization^13^. The ancient construction practices prominently utilized the binders such as clay, mud, limestone, volcanic ash and gypsum in the preparation of mortar since the ancient times, following its setting and hardening properties. Archaeological evidences has shown the usage of Lime started before the prehistoric period in Iran and China^14^. Romans and Egyptians were known for their use of pozzolanic elements, gypsum and quick Lime in early construction, unfortunately the plasters prolonged to set at conventional period of time. Hence the historic engineers and masons experimented with the locally available organic substances like gum Arabic, animal glue, blood of animals, figs, egg whites and yolks, casein, keratin that served as adhesives substances to enhance the weak mortars^15^. Francesco milizia^16^ reported on mixture added with oil to enhance the mortars' impermeability used in aqueducts and cisterns. The carbohydrate-based materials such as sugar, molasses, malt, drum stick gum and arabica gum were incorporated during production to quickly react to form calcium saccharate, which improved the cohesion properties and resistance against environmental forces^17–21^. The cactus (nopal juice) extracts were commonly added to mortars in ancient Mexican buildings to resist the water penetration and crack formation on the surfaces by improved mechanical properties due to the cactus' polysaccharide mucilage interaction with portlandite at early stages^22^. Calcium oxalates (Weddellite, whewellite) are important biominerals found in nature and are the most abundant organic minerals category identified in sediments and hydrothermal veins. The genesis of calcium oxalates has been identified in many limestone structures as layers, and they act as a barrier to reduce the deterioration of stones due to weathering action. The effective role of jaggery (unrefined sugars), kadukkai (*Terminalia chebula*), kulamavu (*Persea Macrantha*) in lime mortars have been analyzed, and it has been found that the addition of fermented organics leads to the formation of weddellite in lime mortar, it has dramatically increased physical and mechanical properties^23^. Protein-lime was mainly used in ancient masonry construction, clay sculptures, mural paintings, and wall paintings; it's been found that the addition of albumen can bring a series of physicochemical property changes stickiness, water repellency, air-entraining and antimicrobial etc.^24,25^.The sixth century C.E. sites of Ellora caves, India, reported on earthen/Lime as a binding material in addition to *cannabis Sativa* (Hemp). Daulatabad fort, India reported on the addition of local vegetal fibres into mortar providing the information about the hempcrete production technology during Yadava regime^26^. The use of organic compounds dated back to ancient times and has widely spread across the world to enhance the mortar's strength. The mortar produced with lime putty and cheese adhered extremely well to stone blocks. The most commonly used additives were oxblood, animal fat, dairy products, tree barks, linseed oil, fig juices, lard, eggs, wine, beer, casein and cactus juice etc.^27^. Based on the regional availability a group of herbs rich in carbohydrate, protein and fat were fermented for few days and used for lime mortars production instead of water. During fermentation, the carbon-di-oxide produced will react with Lime for faster hardening and biomineralize calcium carbonate polymorphs^28^. Much ancient text talks about the use of plant and animal extracts in construction. One such preserved palm-leaf manuscript from the Padmanabhapuram Palace museum (Fig. 1), Tamil nadu, India, described the locally available organic herbs into mortar during the construction practices.

The palm leaf manuscripts acknowledged the traditional usages of organics like palm jaggery (Refined sugar), Kadukkai (*Terminalia Chebula*), Neelamari (*Indigofera Tinctoria*), Aloe vera (*Xanthorrhoeaceae*) and eggs during construction. Recent studies have used natural organic additives to slow down and accelerate the setting time and increase the mortar's workability. Modern analytical techniques like GC–MS were highly recommended to identify organic substances like polysaccharides and fatty acids. The 4th-century Macedonian tombs (Greece) wall paintings revealed the addition of plant gums and their pigments in mural wall paintings, detected by ion chromatography^29^. The byzantine tombs pointed out egg white usage in the lime mortar to enhance the mortar's binding and flexural strength, investigated through the HPLC technique^30^. Despite the above-mentioned recent investigations, to our best knowledge, the determination of organic mineral and its effect on Lime was rarely documented, but this study proposes a multi analytical approach to determine the possible bioactive compounds present in the identified herbs as mentioned in the ancient manuscript and to understand its effective role with lime paste through the sophisticated methods like Gas chromatography and mass spectroscopy (GC–MS), X-Ray diffraction, Fourier transform-infrared spectroscopy, U.V. spectrophotometer, FE-SEM and quantitative methods like protein and fatty acid test methods. The characterized results will help Archaeology department, conservation engineers, architects and N.G.O.s working in the field of conservation to understand the traditional production technology and to produce the simulation mortar to restore the historic structures.

## Experimental section

### Collection of organic herbs

**Figure 2:**
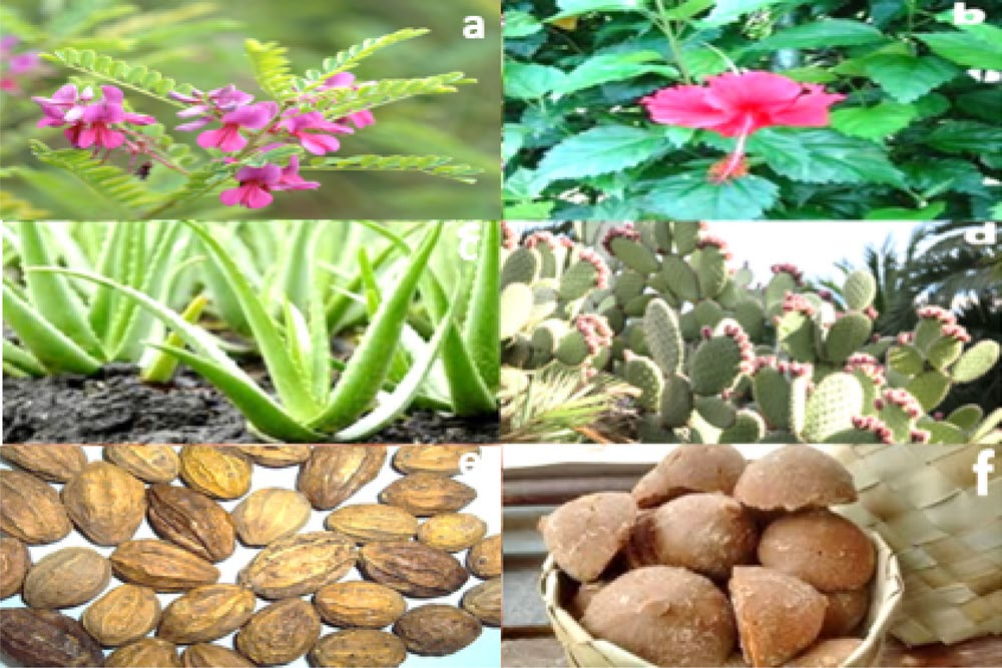
Seeds and plants used in Palace construction, (**a**) Neelamari plant (**b**) Hibiscus flower (**c**) Aloe vera (**d**) Cactus (**e**) Kadukkai seeds (**f**) Palm jaggery.

According to the palm leaf inscriptions and a semi-formal survey among the local people, the locally available healthy plant herbs like Kadukkai, Neelamari, Aloe vera, Hibiscus and Palm jaggery, etc. collected for the experiment, as shown in Fig. 2. The plants were washed with running tap water to remove the organic dust particles on the surface and open air-dried. Further, the dried leaves were gently crushed through pestle mortar to a fine powder. 5% by weight of each herb was soaked in the normal water for 14 days for fermentation. Later the aqueous fermented liquid is filtered through Whatman filter paper 42 for lyophilization^31^ (Blaško et al., 2008; Nandagopalan 2015).

### Quantification of CO_2_ from fermented extracts

The amount of CO_2_ release from 0 to 14 days of fermented organics was regularly measured at an interval of 24 h using a wireless CO_2_ sensor operated by PASCO PS-3208. The sensing element, a pyroelectric device with a quartz reflector at one end of the probe and the other end is the I.R. band filter, absorbs the CO_2_ Gas in the probe. The concentration of CO_2_ is measured by non-dispersive infrared technology (N.D.E.R.) with an operating temperature of 0-50º C, the relative humidity of 0–95%, and the detection limit of CO_2_ concentration ranged between 100 and 1,00,000 ppm^32^.

### Lyophilization of fermented samples

From supplementary Fig. 1. The filtered aqueous extract is being defrizzed at -80ºC and been lyophilized for 24 h. The aqueous sample gets modified into pellet form through the lyophilization process by vacuuming at -50 ºC to evaporate the substance's total moisture. The samples are then extracted through the ethyl acetate in the volume of 1:4 to examine the bioactive compounds through (GC–MS) gas chromatography-mass spectroscopy (Abdurahman, 2019; Degani 2015; Lluveras 2010; Nandagopalan et al., 2015).

### GC–MS analysis

All the 5-ethyl acetate extracted organic samples were injected in an Agilent 7890 A GC system coupled with an MS 5975 selective detector (Agilent Technologies) with a single quadrupole mass spectrometer PTV injector at National Institute of Ocean Technology (N.I.O.T), Pallikaranai, Chennai, Tamil Nadu. The GC–MS operating conditions were set with oven temperature at 50 ºC for 2 min, then 100ºC at 10 ºC/min and finally increased to 200 ºC and been held isothermally for 10 min. The sample injected was 2µL, and the gas carrier was helium with a flow rate of 1 mL/min. All five samples are ionized by electron energy 70 eV. The GC–MS's total running time was 63 min to characterize the organic compounds through the N.I.S.T. 14.L library^18,31,33,35^ . The detection (L.O.D.) and the quantification (L.O.Q.) limits of fatty acids, sugars, lipids, tannins, protein (amino acids) and carboxylic acids were calculated at a significance level of 0.05 and used for the quantitative evaluation of each compounds^29,33^.

### Process of organic lime putty

**Figure 3:**
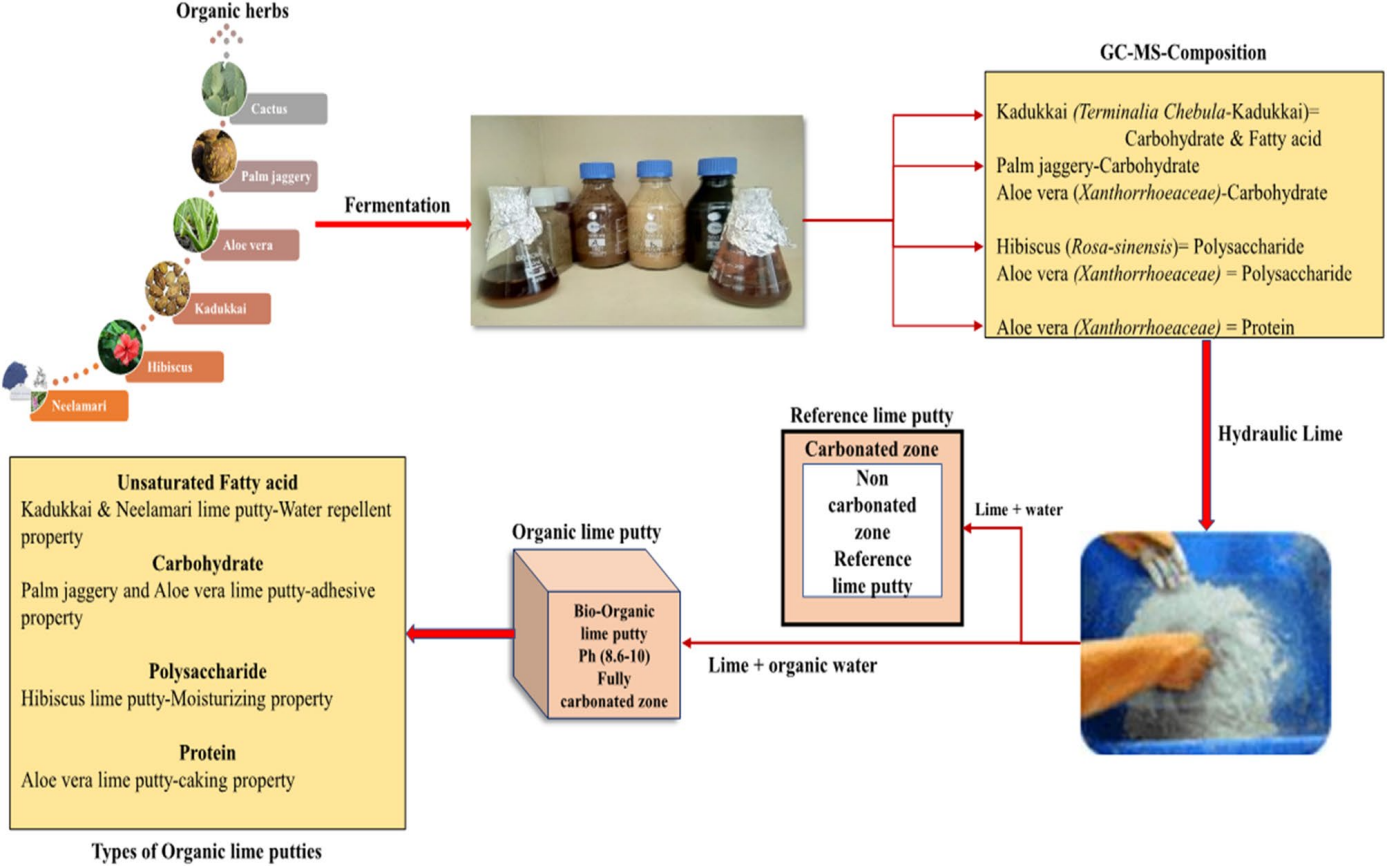
Process of organic lime putty.

Figure 3 Laboratory scale processing of natural herbs followed by anaerobic fermentation for a period of 14 days to extract the bio precipitated solution. The extracted solution was experimented under GC–MS and classified based on its composition namely fatty acids, carbohydrate, polysaccharide and proteins. The fermented organic extract is filtered and mixed with hydraulic Lime (NHL 2.5) procured from Dhone, Andhra Pradesh, to produce the lime putty. Table 1 showed that 1:0.65 as water to Lime (W/L) ratio for reference lime putty and organic water to lime ratio at 1:0.68 (O/L) for Kadukkai, Neelamari, Aloe vera and 1:0.74 for Jaggery and Hibiscus according to flow table test as per I.S. 6932-Part (VIII)-1973 for the right consistency and the free water content of lime putty is maintained between 45 and 70% according to BS EN 459-Part 1^36^.

**Table 1:** Proportion of organic lime putties.

Proportions of organic lime putties	
1	Lime + water (W/L)- (1:0.65) = Reference lime putty
2	Lime + Fermented organic water (O/L) -(1:0.68) (Kadukkai lime putty)
3	Lime + Fermented organic water (O/L) -(1:0.74) (Hibiscus lime putty)
4	Lime + Fermented organic water (O/L) -(1:0.68) (Aloe vera lime putty)
5	Lime + Fermented organic water (O/L) -(1:0.74) (Palm jaggery lime putty)
6	Lime + Fermented organic water (O/L) -(1:0.68) (Neelamari lime putty)

(W/L): Lime to water ratio, (O/L): Lime to organic water.

### UV spectrophotometer analysis

#### Derivatization of Total sugars

2 mg of the fermented solution is taken into a calorimetric tube, and 0.05 ml of 80% phenol is added. Further, 5 ml of concentrated sulfuric acid is added rapidly, the stream of acid being directed against the liquid surface rather than against the test tube's side to obtain good mixing. The tubes are allowed to stand for 10 min. Then they are placed in a water bath for 10 to 20 min at 25-30ºC. The absorbance of the characteristic yellow-orange colour is measured at the scanning range of 540 nm for galactose, hexoses and pentoses using ultra violet-spectrophotometer^37^ (Hitachi UH 5300, Japan) as shown in Supplementary Fig. 2. (Dubois 1951).

#### Determination of total fatty acids (Fame method)

0.25 g of each fermented extract is taken in a 50 ml round-necked volumetric flask. Add 10 ml of 0.2 N sodium methylate to methanol placed on an electric heater for 15 min to reach 100 °C. The solutions should be clear, usually lasting 10 min. The reaction is complete after 15 min, further removing the flask and the condenser from heat until the reflux stops and adding 1% of phenolphthalein solution to the methanol. Adding 1 N sulphuric acid solution to methanol until the solution is colourless, and add 1 ml more. Fit the condenser and boil for another 20 min. Remove the heat source and cool the bottle under running water. Remove the condenser, add 20 ml of saturated sodium chloride aqueous solution, stir, add 5 ml of heptane, plug the flask and shake vigorously for 15 s. Allow it to settle until the two phases have been separated. Repeat the saturated sodium chloride solution until the aqueous layer reaches the lower end of the flask neck, as shown in Supplementary Fig. 3. UV-Spectrophotometric further observes the processed solution with a concentration level (10–100 μg/ml) with an absorbance wavelength (660 nm)^38^.

### Analytical techniques

The mineralogical phases were determined by X-Ray diffraction (XRD) using the Rigaku smart lab II diffractometer according to the diffraction powder method operating at 9 kW and 9 mA with oriented sample holders in polymethyl methacrylate or Si of diameters 25 and 20 mm depending on the amount of material (2–5 mg). The XRD patterns were obtained by scanning at the rate of 1º/minute from 5º to 90º (2 h) and steps of 0.05º (2 h)^39^. Fourier transform infrared spectroscopy (FT-IR) was performed using the Bruker Tensor 27IR with KBr pellets to measure the samples' energy absorption. 5 mg of finely ground < 75 µm specimen powder was homogenously mixed with 250 mg of KBr powder until the mixture had the consistency of fine flour and then pressed into a thin 15 mm diameter disc with infrared spectra obtained at 4000–400 cm ^− 1^ range, with a resolution of 4 cm^−1^ 32 scans respectively^40–43^. The samples' morphology was observed with a Carl Zeiss Supra 55 FE-SEM. The elemental analyses were performed using an X-ray dispersive spectrometer (E-DAX) resolution of 0.8 nm coupled to F.E.S.E.M. Each sample was coated with gold sputter (90 s) in a vacuum evaporating system. The sample was observed at a magnification range of 3300–10,000 × with low vacuum mode at 20 kV (Izaguirre, Lanas, & Álvarez 2010; Macwilliam & Nunes, 2019; Pradeep & Selvaraj, 2019).

## Results and discussions

### Liberation of CO_2_ & biomineralization of calcite

Fermentation was performed on the herbs to enhance the chemical reactions induced by microorganisms or the enzymes that split the complex compounds into the simplest substances in the absence of oxygen. This splitting of complex compounds is one reason for the carbonation of Lime. The fermentation periods of the organic herbs were fixed according to greater CO_2_ levels. The air bubbling in the airlock container will gradually slow down, becoming more and more infrequent and then stopping up altogether^44^ (Reddy 2018).

**Figure 4:**
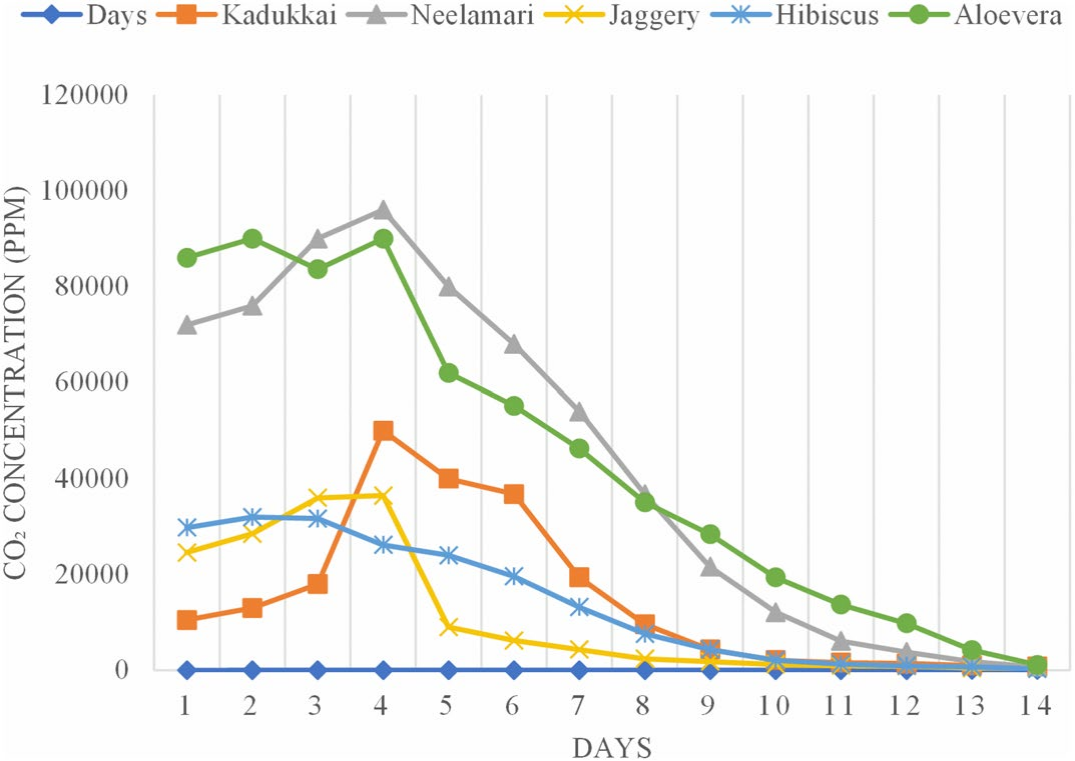
CO_2_ Quantification.

The amount of liberation of CO_2_ ranged between 10,000 and 90,000 ppm during the complete fermentation process shown in Fig. 4. Organic herbs like neelamari and aloe vera have significantly released the highest CO_2_ of 96,000 and 90,000 ppm on the 4th day of fermentation. Further, it slowed down its activity as the days progressed. Similarly, the palm jaggery and kadukkai have liberated the considerable amount CO_2_ of 36,500 and 50,000 ppm on the 4th day on signalling the rise of air bubbles from the bottom, indicates the complete ethanolic transformation activity. But the hibiscus has rapidly liberated the CO_2_ of 32,000 ppm on the 2nd day and moderately slowing down the emission on the 7th day, indicating the hibiscus has pyruvate into complete alcoholic transformation by splitting the complex compounds into sugars such as glucose, fructose and sucrose products. The fermentation measurements suggested, the liberation of CO_2_ was found high for neelamari and aloe vera, followed by kadukkai and jaggery; hence all these four organic fermented extracts can be incorporated into the lime mortar on the 4th day to activate the internal carbonation and to strengthen the mortar due to the internal reaction between the lime particles and organically induced CO_2_ to improve the calcite crystals. But the hibiscus herb showed high liberation on the 2nd day and gradually slowed down the activity, indicating it took 2 days to ferment and extended its transformation till 14 days.

Moreover, the fermentation days have significantly varied according to the organic herbs' phytochemical compounds; hence the number of days for fermentation was not kept constant in this study^32^. Liberation of CO_2_ is very important for setting and hardening of lime mortars in heritage buildings. In general, lime sets by carbonation by absorption of CO_2_ from the atmosphere to precipitate the calcium carbonate (Eq. 1).

1 $${\mathrm{CaO }} + {\mathrm{ Atmospheric}}\,{\mathrm{CO}}_{2} = {\mathrm{ CaCO}}_{3}$$

Since the lime mortars, walls are huge and a few meters thick, the outer portion up to few centimeters gets carbonated because of atmospheric carbon dioxide. However, the internal core mortars remain un-carbonated for decades and prolong the lime mortars' hardening and resulting in slow strength gain. This main drawback of slow setting and strength gain is enhanced by fermented extract into Lime instead of water. The internal supply of CO_2_ results in the precipitation of calcium carbonate in bio calcite and metastable aragonite and vaterite^42–49^. The biomineralization of calcite from CO_2_ supplied from organic extract helps in fast setting and increased load-bearing capacity of heritage structures. Hence in the organically modified mortars, the hydrated phased are due to atmospheric and internal carbonation due to organics. The solubility of calcium compounds influences the degree of carbonation as the solubility of Ca(OH)_2_ being about 100 times higher than that of all three CaCO_3_ polymorphs. Therefore, self-healing will preferentially occur in partially carbonated lime mortar, where the uncarbonated part will provide Ca (OH)_2_ to fill thin cracks, thereby enhancing durability. Furthermore, peaks of all three different polymorphs of CaCO_3,_ namely vaterite, aragonite and calcite, are found in the XRD pattern. This combination influences the dissolution and the subsequent recrystallization process and thereby contributes to the mortars' self-healing nature, making the mortars more durable^39,49,50^.

### GC–MS analysis of fermented extracts

The 3% organic fermented extracts were screened under the GC–MS Agilent 7890A G.C. system with an MS 5975 selective detector; it detected the various bioactive compounds present in the five fermented extracts unsaturated fatty acids polysaccharides, carbohydrates and proteins substrates. The complete phytochemical compounds detected from the GC–MS analysis is shown in Table 2.

**Table 2:** Phytochemical compounds obtained from GC–MS chromatography analysis.

Identified bio-active compounds (fatty acids, carbohydrates & protein)																				
Organic Herbs	Fatty acids											Organic compound		Organic acids		Carbohydrates				Protein
	Hexadecenoic acid	Octadecanoic acid	Pentadecane	oleic acid	Pentadecanoic acid	Eicosanoid acid	Cholestane	Docosatetraenoic acid	Docosonaic acid	Cyclododecane	Pentafluoronoic acid	Styrene	furancarboxaldehyde	Oxalic acid	Fumaric acid	Glucopyranoside	Mannopyranose	Galactose	Glucose	Thiophene
*Terminalia Chebula (Kadukkai)*	+ + +	+ + +	+	−	+ +	+	−	+ +	+ +	−	+	+	+	−	−	−		−	−	+
*Palm jaggery*	−	−	+	−	−	−	−	+ +	−	+ +	+	−	−	−	−	+ +	+ +	+ +	+ +	−
*Indigofera tinctoria Neelamari*	+	+	+	+	+	−	+	−	+	+	−	−	−	−	−	−	-	−−	−	−
*Hibiscus Rosa sinensis*	+ +	+ +	+ +	−	+ +	−	+ +	−	+ +	−	+ +	−	−	+ +	+ +	−	−	−	−	−
*Xanthorrhoeaceae (Aloe vera)*	+	+	+	−	+	−	−	+	−	−	+	+ +	−	−	+ +	+	+ +	+ +	+ +	+ +

+ ** +  + **, very abundant: (> 40%) + ** + **, abundant: (15–40%) + , Scarce + **/**−.

#### GC–MS unsaturated fatty acid detection

The ethyl acetate extracted fractions of organic fermented samples revealed the major bioactive compounds, interpreted through the GC–MS NIST 14. L. The representation of bioactive compounds is tabulated with peak concentration (%), retention time (R.T.), molecular formula, molecular weight and weight percentage with 100 mg/ml. Tables 3, 4, 5, 6, and 7 and supplementary method 4 fermented extracts namely kadukkai (*T. Chebula)* resulted in the form of unsaturated fatty acids compounds like citric acid, hexadecenoic acid, trans-octadecanoic acid, 13-octadecanoic acid and 6-octadecenoic acid with the overall unsaturated fatty acid percentage quantified around 54.1%. Neelamari (*Indigofera tinctoria)* chromatogram fractioned adipic acid, demecolcine, nalmefene, strychnine, cholestane-6-one, aspidodasycarpine, cyano, rhodopin, lycopene, phosphine with 80.78% in 100 mg/ml concentration^34^ (Nayar 1999). Palm jaggery (refined sugar) depicted the acetamide, lumicolchicine, pentadecanedioic acid, acetic acid, valine (amino acid), cyclodecasiloxane, pentamide and manopyranose content around 92.85%. The high-level acetic acid observed in palm jaggery reacts with the lime particle forming calcium acetate to promote the hydration properties due to the carboxyl groups to enhance the initial strength and internal voids the mortar^46,52–54^. Sucrose is the most common sugar, made up of α-glucose and β-glucose linked together by a glycosidic bond. The hydroxyl groups make the molecule highly water-soluble. As far as the lime traditions are concerned, the use of sugars has been documented in historic lime mortars in various countries where its interaction with C–S–H has been observed by adsorbing the Ca (OH)_2_ to increase the binding properties of the mortar. The addition of jaggery to Lime increases the solubility of Lime in water by 84%. Hence, instead of water jaggery extract, water can be added during slaking. It helps in more conversion of calcium oxide (Lime) into calcium hydroxide (portlandite). Jaggery contains 60–85% sucrose, 5–15% glucose and fructose, 0.4% protein and 0.6–1% of minerals^9^. Hibiscus (*Rosa Sinensis)* contained hexadecenoic acid, 14-octadecadienoate, tetracontane, fumaric acid (pigment), tritriacontane, 9-octadecene and hentriacontane ranging around 8.37%, resulting in a very low unsaturated fatty acid from the chromatogram. Table 11 Aloe vera (*Xanthorrhoeaceae)* recorded the pentanoic acid, tetradecanoic acid, n-hexadecenoic acid, oleic acid,9-octadecanoic acid and docosanoic acid around 47.72% from the chromatogram^22,53^. The reduced unsaturated fatty acid was observed due to the polysaccharide presence (starch, cellulose and glycogen) in hibiscus organic fermented extract shown in Table 12^17,31^. The higher unsaturated fatty acid was found in palm jaggery, followed by T. chebula and aloe vera fermented extracts, in agreement with CO_2_ quantified liberation observed. The role of unsaturated fatty acid compounds in the fermented extracts interacts with the lime particles to improve the water-resistance, mechanical strength and enhance the microstructure's formation. Hence the addition of unsaturated fatty acids have at least one double bond between carbon atoms, and they are thus more reactive and produces compact mortar (Zhang, & Zheng, 2014; Rampazzi et al., 2016).

**Table 3:** Phytochemical components of *Terminalia Chebula* (kadukkai) ethyl acetate fraction by GC–MS.

Sl no	R.T (min)	Name of the compound	M.F	M.W(g/mol)	P.A (%)
1	29.896	Citric acid	C_6_H_8_O_7_	192.12	6
2	47.88	Hexadecenoic acid	C_16_H_32_O_2_	256.43	11.21
3	51.41	Trans-octadecanoic acid	C_18_H_36_O_2_	284.48	4.66
4	51.61	Octadecenoate	C_20_H_38_O_2_	310.51	5.37
5	51.845	13-Octadecenoic acid	C_18_H_34_O_2_	282.5	10.15
6	53.329	6-Octadecenoic acid	C_18_H3_4_O_2_	282.5	16.71

**Table 4:** Phytochemical components of *Palm jaggery* Alditol fraction by GC–MS.

Sl no	R.T (min)	Name of the compound	M.F	M.W(g/mol)	P.A (%)
1	4.89	Acetamide	C_2_H_5_NO	59.07	12.76
2	6.14	Lumicolchicine	C_22_H_25_NO_6_	399.04	15.19
3	6.67	Pentadecane dioic acid	C_15_H_28_O_4_	272.38	3.93
4	7.69	Acetic acid	CH_3_COOH	60.05	46.53
5	7.81	Valine (amino acids)	C_5_H_11_NO_2_	117.15	6.32
6	8.08	Cyclodecasiloxane	C_20_H_60_O_10_SI_10_	741.53	1.56
7	8.47	Pentamide	C_5_H_11_NO	101.15	2.25
8	8.72	Manopyranose	C_6_H_12_0_6_	180.15	4.31

**Table 5:** Phytochemical components of *Indigofera tinctoria* ethyl acetate fraction by GC–MS.

Sl no	R.T (min)	Name of the compound	M.F	M.W(g/mol)	P.A (%)
1	46.47	Adipic acid	C_6_H_10_0_4_	146	0.26
2	47.33	Demecolcine	C_21_H_25_NO_5_	371	0.17
3	48.31	Nalmefene	C_21_H_25_NO_3_	339	1.16
4	51.68	strychnine	C_21_H_22_N_2_O_2_	334	0.43
5	56.55	Cholestan-6-one	C27H_46_O_3_	418	63.69
6	59.24	Aspidodasycarpine	C_21_H_26_N_2_O_4_	370	1.73
7	59.65	Cyano	C_3_H_3_NO_2_	85	4.33
8	60.06	Rhodopin	C_40_H_58_O	554	13.57
9	61.03	Lycopene	C_40_H_56_	536	0.033
10	62.69	Phosphine	PH_3_	34	10.34

**Table 6:** Phytochemical components of *hibiscus rosasinensus* ethyl acetate fraction by GC–MS.

Sl no	R.T (min)	Name of the compound	M.F	M.W(g/mol)	P.A %
1	36.1	Hexadecenoic acid	C_16_H_32_O_2_	256.43	0.069
2	37.11	n-Hexadecenoic acid	C_16_H_32_O_2_	256.43	0.186
3	40.03	14-octadecadienoate	C_18_H_32_O_2_	280.4	0.146
4	48.44	Tetracontane	C_40_H_82_	560.03	0.039
5	50.3	Fumaric acid	C_4_H_4_O_4_	116.07	0.521
6	50.32	Tritriacontane	C_43_H_88_	605.17	1.785
7	53.02	9-Octadecene	C_18_H_38_	254.5	2.085
8	56.72	Hentriacontane	C_31_H_64_	436.85	3.542

**Table 7:** Phytochemical components of Aloe vera (*Xanthorroeaceae)* ethyl acetate fraction by GC–MS.

Sl no	R.T (min)	Name of the compound	M.F	M.W(g/mol)	P.A (%)
**Bioactive molecules of Fermented aloevera sample**					
1	3.455	Pentanoic acid	C_5_H_10_O_2_	102.13	4.82
2	32.15	Tetra decanoic acid	C_14_H_28_O_2_	228.37	1.03
3	37.18	n-Hexadecenoic acid	C_16_H_32_O_2_	256.43	22.22
4	41.12	Oleic acid	C_18_H_34_O_2_	282.47	0.18
5	41.65	9-Octadecanoic acid	C_18_H_36_O_2_	289.48	16.2
6	63.28	Docosanoic acid	C_22_H_44_O_2_	340.56	3.27

**Table 8:** Relative percentage of sugars and fatty acid in *Xanthorrhoeaceae leaf* (aloevera).

R.T (min)	Name of the compound	M.F	M.W(g/mol)	P.A (%)
15.75	4,7-Dihydroxyl phenanthroline	C_12_H_8_N_2_O_2_	212.2	3.14
18.16	9-H-Purine	C_5_H_4_N_4_	120.11	3.39
18.41	Galactitol, Hexacetate	C_6_H_14_O_6_	182.17	3.13
18.58	Acetic acid	CH_3_COOH	60.05	3.55

#### GC–MS polysaccharide detection

The assessment of polysaccharides (starch, cellulose and glycogen) through GC–MS analysis of the fermented organic herbs resulted in showing the sugars and fatty acid bioactive compounds from Tables 8, 9, 10, 11, and 12 and supplementary method 4. The fermented aloe vera organic extract revealed galactitol, hexacetate and acetic acid at 13.21% in 100 mg/ml concentration. Palm jaggery recorded the presence of beta-d-galactopyranose, beta-d-mannopyranose and iditol (galactokinase) at 11.80% and fatty acids of 83.02%. Palm jaggery, which is predominantly sucrose, upon reacting with calcium oxide and silica in clay, forms strong bonds and hardens on drying. Lime mortar made with fermented jaggery extracts has decreased the segregation and bleeding properties. Sugar is a carbohydrate substance composed of carbon, oxygen, and hydrogen. It mostly binds the intermolecular compounds to strengthen the mortar from cracks and acts as a load-carrying element for the bedding mortar^54^. Aloe vera phytochemical analysis screened 13.21% of carbohydrate and polysaccharides, which disperses in water, often making a hydro-colloidal system and retain water. Therefore, aloe vera extract's main function is water-retention, exhibiting quick frying and prevents shrinkage crack on the render after hardening. In addition, the viscosity of the extract improves the plasticity of the mortar by applying thin layers of polished finish on the surfaces. Converting into ethanolic fermented extract reduces the specific surface area and pore distribution system on hydrated phases (Chattopadhyay, 2013). The sugars peaks were untraceable in the further analysis, which responded with active fatty acid compounds in kadukkai, neelamari and fermented hibiscus extracts. Kadukkai extracts revealed citric acid (6%), hexadecenoic acid (11.21%), octadecenoate (5.37%), 13-octadecenoic acid (10.15%) and 6-octadecenoic acid (16.71%) as shown in Table 10 with the detection percentage ranged around 54.1%. Similarly, the neelamari and hibiscus also fractioned the fatty acid peaks like cyclodecasiloxane (9.15%), methyl hexadecadienoate (10.86%), oleic acid (26.54%), octadecadienoic acid (8.96%) as shown in Table 11 with weight percentage detected around 72.53%. Hibiscus extract resulted in high fatty acid compounds like pentane dioic acid (6.47%), tridecanoic acid (5%), octadecadienoic acid (4.65%), hexadecatrienoic acid (4.99%), octadecenoate acid (4.9%), 9-octadecanoic acid (15.18%), octadecanoic acid (8.14%) with overall percentage detected around 86.34% as shown in Table 12. The unsaturated fatty acid concentration levels in three fermented extracts has indicated the role of high hydrophobicity to the lime hydrate phases on creating the air entrapment to slow down the carbonation activity and also acts as a water-repellent matrix by reducing the drying shrinkage cracks on the structures^56^ (Hrdlickova kuckova 2015; Rao et al., 2015).

**Table 9:** Relative percentage of sugars and fatty acids in *palm jaggery*.

Sl no	R.T (min)	Name of the compound	M.F	M.W(g/mol)	P.A (%)
1	3.662	Iproniazid	C_9_H_13_N_3_O	179.21	21.11
2	11.36	Cylododecanecarboxylic acid	C_13_H_24_O_2_	212.33	12.66
3	11.81	2,610,10-Tetramethyl-1-oxaspiro	C_13_H_20_O	192.3	10.75
4	15.53	4,7-Dihydroxy -1,10-phenanthroline	C_12_H_8_N_2_	180.21	22.84
6	16.82	Phenol	C_6_H_5_OH	94.11	5.56
8	17.11	Beta-D-Galactopyranose	C_12_H_22_O_11_	342.3	4.133
9	17.26	Beta-d-Mannopyranose	C_6_H_12_0_6_	180.16	7.67
10	18.17	Pyrrolidine	C_4_H_9_N	71.12	2.8
11	18.42	Iditol, Hexacetate	C_6_H_14_0_6_	182.17	8.1

**Table 10:** Relative percentage of fatty acids in Kadukkai *(T. chebula).*

Sl no	R.T (min)	Name of the compound	M.F	M.W(g/mol)	P.A (%)
1	29.896	Citric acid	C_6_H_8_O_7_	192.12	6
2	47.88	Hexadecenoic acid	C_16_H_32_O_2_	256.43	11.21
3	51.41	Trans-octadecanoic acid	C_18_H_36_O_2_	284.48	4.66
4	51.61	Octadecenoate	C_20_H_38_O_2_	310.51	5.37
5	51.845	13-Octadecenoic acid	C_18_H_34_O_2_	282.5	10.15
6	53.329	6-Octadecenoic acid	C_18_H3_4_O_2_	282.5	16.71

**Table 11:** Relative percentage of fatty acids in Neelamari (*Indigofera Tinctoria*).

Sl no	R.T (min)	Name of the compound	M.F	M.W(g/mol)	P.A (%)
1	48.15	Cyclodecasiloxane	C_20_H_60_O_10_Si_10_	741.5	9.15
2	49.13	Methyl-8-Hexadecadienoate	C_17_H_30_O_2_	266.4	10.86
3	51.41	Methyl-8-Octadecadienoate	C_17_H_30_O_2_	294.5	6.2
4	52.78	Cyclodecasiloxane	C_20_H_60_O_10_Si_10_	741.5	10.82
5	53.09	Oleic acid	C_18_H_34_O_2_	282.47	26.54
6	57.42	9,12- Octadecadienoic Acid	C_18_H_32_O_2_	280.4	8.96

**Table 12:** Relative percentage of fatty acids in Hibiscus.

R.T (min)	Name of the compound	M.F	M.W (g/mol)	P.A (%)
15.33	Butane dioic acid	C_19_H_34_O_2_	294.5	6.64
16.44	1,2,5-Oxadiazole	C_3_H_2_N_2_O_3_	114.06	7.5
21.09	Methyl phosphonic acid	CH_5_O_3_P	96.02	6.18
30.98	Pentane dioic acid	C_5_H_8_O_4_	132.12	6.47
39.43	Tridecanoic acid	CH_39_(CH_2_)_11_ COOH	214.34	5
48.48	3-Methyl benzoic acid	C_8_H_8_O_2_	136.15	5.68
49.3	2,6-Di-fluro 3-methylbenzoic acid	C_8_H_6_CL_2_O_2_	205.03	11.01
51.4	9,12- Octadecadienoic acid	C_18_H_32_O_2_	280.44	4.65
51.6	10,13- Hexadecatrienoic acid	C_16_H_28_O_2_	252.39	4.99
51.78	9- Octadecadienoate acid	C_19_H_34_O_2_	294.5	4.9
53.18	9-Octadecenoic acid	C_18_H_34_O_2_	282.47	15.18
53.68	Octadecanoic acid	C_18_H_34_O_2_	282.47	8.14

The unsaturated fatty acids are the components that have imparted the hydrophobicity to the lime paste because it comprises the highest amount of C–C double bonds. The organics herbs with more amount of monounsaturated fatty acids (oleic acid) are more effective in imparting the hydrophobicity. The recent study on the addition of oleic acid to the lime paste has observed decreased particle size precipitates^57–60^. The morphology of kadukkai lime paste, aloe vera lime paste, neelamari lime paste mixes has indicated the transformation of calcium hydroxide in forming the carbonate polymorphs at 7 days of testing. The unsaturated fatty acids like citric acid, hexadecenoic acid, trans-octadecanoic acid, 13-octadecanoic acid and 6-octadecenoic acid present in fermented plant extracts have accelerated the formation of microstructure at an early stage and play a role in biological nucleation.

Moreover, it regulates the growth of calcium carbonate crystals and leads to smaller particles and a more compact structure. The lime paste subsumed with unsaturated fatty acids has better mechanical properties, water resistivity and weather resistance than common reference lime paste. (Ziyang Liu 2016).

The functions of protein in lime paste include air-entraining, decrease roughness, viscous enhancing performance and environmentally friendly admixtures for modern construction materials. The addition of amino acids (valine) has improved lime paste's performance due to the interfacial activity of the protein-peptide chains and the bio-mineralization effect. Fang et al. discussed that proteinaceous substances' addition improves the consistency, cohesiveness, durability and reduces the risk of cracking of the lime mortar. Simultaneously, it has a negative influence on the compressive strength and surface hardness of the mortar along with much more compact and slows down the carbonation progress of the lime mortar^14^. Yang and Zeng et.al. Revealed that the presence of carbohydrates played a crucial role in the microstructure and consolidation of lime mortars. Carbohydrates act as a template and consolidate the loose structures and result in strength improvement and durability^23^. Thirumalini et al., in their research work they have analyzed the influence of organically added lime mortar on mechanical and physical properties of lime mortar and revealed that the addition of organics has enhances the compressive strength of lime mortar due to the reduction of a higher amount of larger pores into micropores. The addition of organics in the form of carbohydrates and protein in the lime mortar mix would have also prolonged the reaction and retarded the initial growth to arrest the drying shrinkage cracks on the lime paste.

The similar presence of calcite and vaterite in the mortar samples with traces of calcium oxalates (weddellite) could contribute to the self-healing of lime mortar in enhancing the crystalline growth high carbonation rate^42,46^. From the overall results, the addition of organics has proven that phytochemicals have altered the mineralogical formation and significant advantages such as setting acceleration, water repellency, hydrophobicity, strength enhancement and carbonation rate to produce an eco-friendly bio-organic mortar.

This study's phytochemicals investigation has contributed to understanding the interaction mechanism between the lime particles and fermented organics. Although many efforts have been devoted to identifying the original components of the ancient building materials, it is not always possible to find the exact reason for the structures' survivability and longevity. This is especially true for the organic additions, either because they were incorporated in small amounts (below 5%), thus falling short on detecting the possible interaction and type of phytochemicals that have improved the required structural aspects^56^.

#### Quantification of fatty acid (Fame Method)

The fatty acid methyl esters (FAME) detected the fatty acid esters derived from the transesterification of fats in the presence of methanol and been screened with a U.V. spectrophotometer between 290 and 660 nm. The absorbance produced the concentration of three herbal extracts that was plotted through the linear regression modelling coefficient (> 0.9981) and calculated the limit of quantification (L.O.Q.) and limit of detection (L.O.D.) ranging around 0.026 and 0.088 shown in Supplementary Figs. 5 and 6^12,20,29,33^.

2 $$LOQ \, = 10S_{a} /b_{1}$$

3 $$LOD \, = \, 3S_{a} /b_{1}$$

Sa: Standard deviation of the response, b: slope of the calibration curve.

The fatty acid concentration of kadukkai was 32.91 µg/ml, neelamari and aloe vera around 154.43 and 383.54 µg/ml through the fatty acid methyl esters transesterification method. De architectura by Vitruvius stated that the natural oil and plant extracted fatty acids were among the most common types of water-repellent additives used in ancient mortars for coating in antiquity stucco works. It's highly reactive because it contains a high amount of Oleic acid (26.4 wt%), hexadecenoic acid (22.22 wt%) and octadecenoic, commonly called (linolenic acid) (10.86 wt %) with two and three double bonds, in agreement with the GC–MS chromatograms as shown in Tables 2, 3,4, and 5 and release of high carbon dioxide was similarly detected through the fermented CO_2_ quantification analysis shown in Table 2^11^. The unsaturated fatty acid like tung oil, olive oil, plant extracted oil, animal glue and casein are the most promising additive for improving the repair mortar durability and hydrophobicity to the molecular structure of the mortar. The addition of fatty acid components improves the binding capacity among the grains of lime particles due to their complex fatty acid chain structure. The past reported literature stated that plants contain fatty acid molecules such as oleic acid and stearic acid. Linoleic acid and minor ester fractions have provided the impervious layer to the surfaces by preventing structure instability. The presence of an appreciable number of di-unsaturated esters of linoleic acid is prone to polymerization reaction when exposed to the outer atmosphere. Drying could be rigid the molecular structure with an increase in mechanical strength^17,56,58–60^.

#### UV spectrophotometer total sugar regression modelling

The Beer lambert law is a linear relationship between the absorbance and the concentration, molar absorption coefficient and optical coefficient of a solution.

4 $$A \, = \, \varepsilon \, c \, l$$

A: Absorbance, ε: Molar absorption coefficient M cm^-1^, c: Molar concentration M, l: Optical path length cm.

The U.V. spectrophotometric quantification analysis recorded total sugars through the phenol sulphuric reactive solution with the concentration level of (100-500 µg/ml) to evaluate the absorptivity and the wavelength detection limit ranging between (540 nm). Tables 8 and 9 indicated that total sugars were highly observed in palm jaggery kadukkai and aloe vera solutions around 6867.09 µg/ml, 424.05 µg/ml and16.46 µg/ml and with an absorbance level of 5.425. 0.335 and 0.329, respectively^37^. Further reagents like neelamari and hibiscus have recorded a moderate sugar concentration level due to unsaturated fatty acid in agreement with the GC–MS analysis performed on the fermented extracts. The GC–MS polysaccharide analysis detected the glycosides, monomers, monosaccharides, mannopyranose, glucopyranose and D-glucose in palm jaggery and aloe vera around 3.13 µg/ml and 11.17 µg/ml as shown in Tables 8 and 9, with > 1% sugar traced in other fermented herbs. For the realization of the calibration curve, it worked with the absorbance values obtained, the correlation coefficient (r = 0.998) and y-intercept of 0.079 × with LOQ = 0.013 and LOD = 0.067 was found to be statistically significant when performing the hypothesis testing on total sugars by UV-spectrophotometry as shown in supplementary Fig. 5 The sucrose level accelerates the lime mortar's initial hardening to significantly improve weather resistance by many folds. The addition of proteins acts as an air-entraining agent in fresh mortars and increases workability. It also acts a waterproof to mortar and controls the water movement. The basic reason for adding the proteinaceous additives is that they form a thin layer over the lime particles and slow down the hydration process. Further, calcium ion formation will increase the solubility on discouraging calcium hydroxide formation from strengthening the mortar^18,37,54^.

### X-Ray diffraction (XRD) interpretation

**Figure 5:**
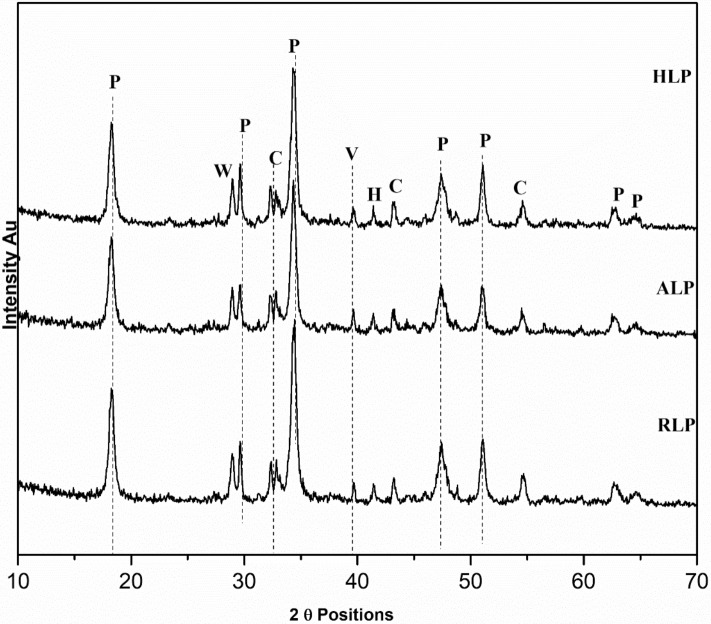
7th day XRD results of organic lime putty—P: Portlandite, V: Vaterite, A: Aragonite, W: Weddellite, A: Aragonite, C: Calcite, H: Hatrurite. HLP: Hibiscus lime putty, ALP: Aloe vera lime putty.RM; Reference lime putty.

**Figure 6:**
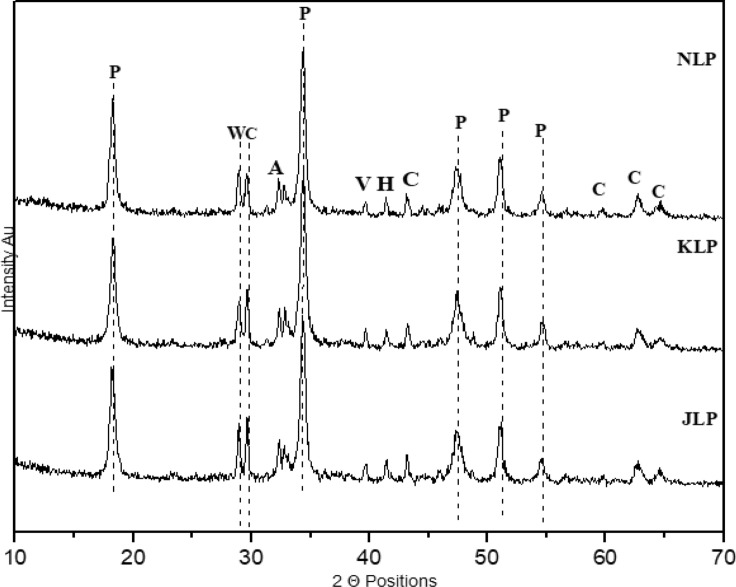
7th day XRD results of organic lime putty—P: Portlandite, V: Vaterite, A: Aragonite, W: Weddellite, A: Aragonite, C: Calcite, H: Hatrurite: Neelamari lime putty, KLP: Kadukkai lime putty, JLP: Jaggery lime putty.

After GC–MS analysis, XRD powder diffraction was performed on organic lime putty samples to evaluate their interaction with fermented herbs. Figures 5 and 6 shows mineralogical phases of the all mortar (reference and organic mortars) with crystalline polymorphs of calcium carbonate (calcite, aragonite and vaterite) and portlandite in confirmation with calcite vibration at 1409 cm−^1^ and portlandite peak at 3641 cm^−1^ in FT-IR analysis. But peaks in organic incised lime putty samples have revealed the overall formation of high portlandite peaks with low-intensity calcite peaks with traces of vaterite and aragonite peaks are observed. Minor traces of hatrurite peaks formed in insoluble silica present in the organic fermented herbs reacting with lime particle forming calcium hydrate silicates observed in K.L.P., JLP NLP organic lime putty samples validated with silicate spectral vibration observed at 420 and 478 cm−^1^. The organics incorporated lime putty samples modified their crystalline formation and properties like workability, water repellency, air entrapment, accelerator, and retarding effect with the interaction of biomolecules present in the fermented herbs^23,28,62^. Likewise, the N.L.P., K.L.P., A.L.P. and H.L.P. samples are rich in the presence of unsaturated fatty acid and fraction of sugars compounds, which has produced an internal dissociation between the lime particles by imparting hydrophobicity, making the surface more porous to increase the carbonation resulting in a gradual increase of calcite peaks with traces of fatty acid FT-IR vibration observed at 3392.1 cm−^1^. The unsaturated fatty acid is mostly prone to hydrophobicity. The formation of calcium oxalates (dehydrated-weddellite) peaks is observed due to oxalic acid (unsaturated acid) in the fermented organic, converting it into calcium oxalates to develop the formation of calcite slowly. The fraction of sucrose, glucose substrates present in the fermented organics tends to form strong bonds between the lime particle to develop crystalline hydrated phases to stabilize the formation of carbonate polymorphs as the days progress. J.L.P., A.L.P. and N.L.P. samples are rich in polysaccharide in agreement with GC–MS analysis. They have improved the early formation of calcite peaks, with possible break down of alcohol and CO_2_ converting the Ca (OH)_2_ to calcium alkoxide and calcium carbonate (Elert, Rodriguez-Navarro 2002). The presence of alcohol influences the metastable carbonate polymorphs phases by stabilizing them to increase the metastable vaterite and aragonite phases. The high intense portlandite peaks are similarly observed in all the organic samples. Additionally, carbohydrate FT-IR vibration peaks at 1583 cm^−1^ confirmed the addition of polysaccharides (starch, glucose and cellulose) would act as water reducers, water retention agents and set retarding effects to the organic incorporated samples^19,21^. This overall inflated transformation of portlandite peaks to calcite in reaction with internal recarbonation activity due to the organic fermented herbs will modify the amorphous phases to develop the crystallinity in forming the hydrated phases as the day's progress can be achieved with better absorbing of external CO_2_ through the external environment.

### Infrared spectroscopy (FT-IR)

**Figure 7:**
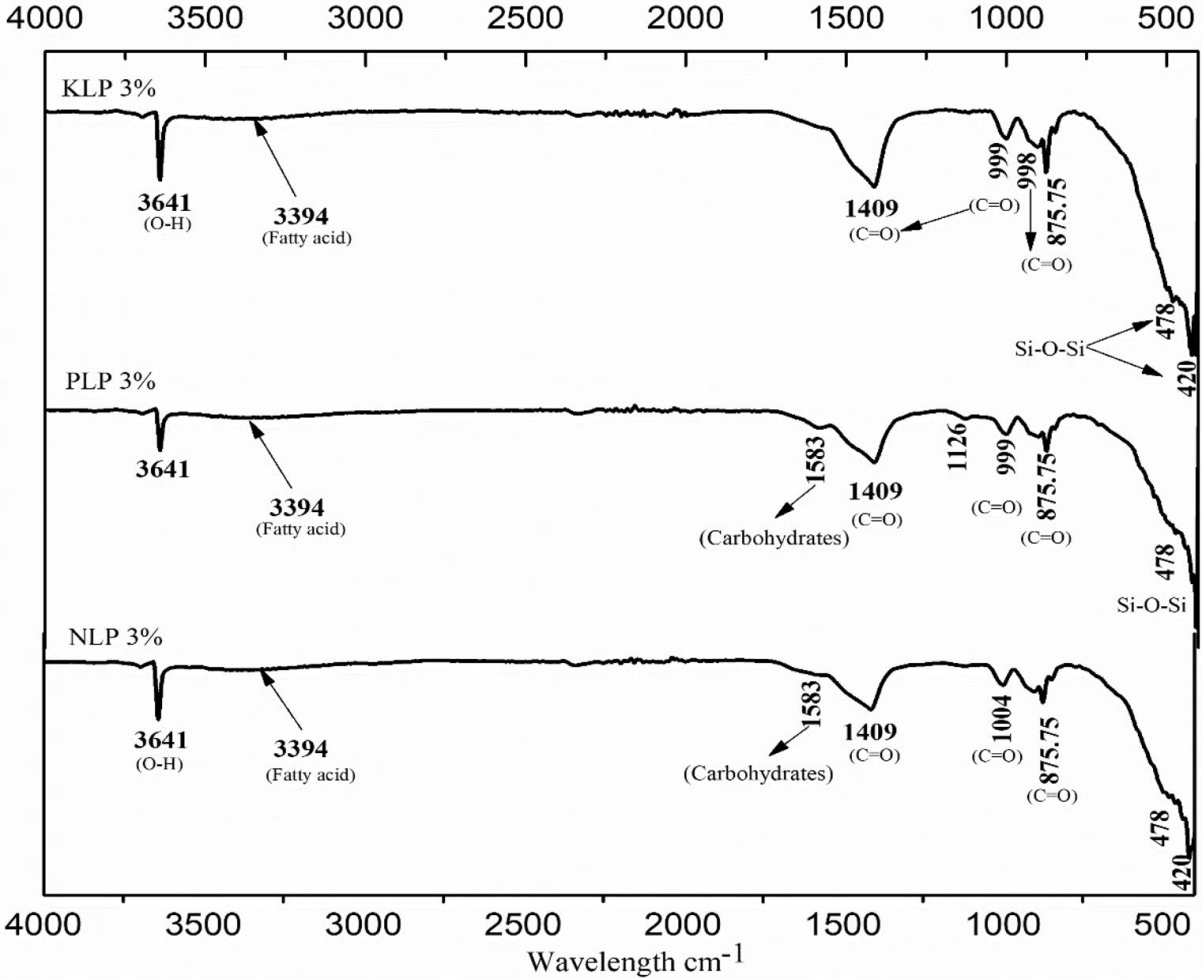
FT-IR organic spectrum of (**a**). Kadukkai lime putty (**b**). Palm jaggery lime putty (**c**). Neelamari lime putty.

**Figure 8:**
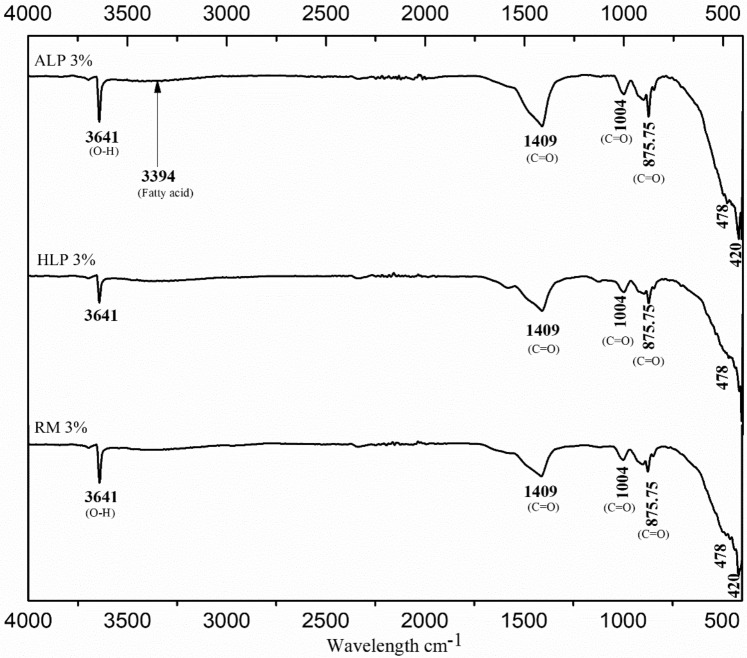
FT-IR spectrum of organic lime putty H.L.P.: Hibiscus lime putty, A.L.P.: Aloe vera lime putty and R.M.: Reference mortar. C = O: Calcite, O–H: Portlandite.

As a confirmation to the mineralogical investigation, FT-IR analysis was performed on the lime putty sample to investigate the organic, polymers and inorganic materials from the sample wavelength, as shown in Figs. 7 and 8. The reference lime putty sample revealed the presence of minor (O–H stretching) with intermolecular H-bond hydroxyl group at 3641 cm^−1^ and (C–H asymmetric stretching) vibration at 1409 cm^−1^,874.15 cm^−1^ (CH_2_ out of plane bending) show the presence of calcite peaks with traces of silicate vibration depicted at 1004 cm^−1^ in agreement with mineralogical investigation^64^. It's been evidenced that all the organically modified lime putty samples have revealed the intense characteristic peak portlandite (O–H stretching) at 3641 cm^−1^ and initially transformed calcite around 1400–1475 cm^−1^ assigned to the in-plane bending and symmetric carbonate out of plane bending vibration of calcite and aragonite. Minor peaks of vaterite have also been confirmed at 999 and 998 cm^−1^ around 900–1080 cm^−1^. The traces of silicate peaks at 420, and 480 cm^−1^ indicate the calcium hydrate silicate formation formed hatrurite peaks (C–S–H) in par with XRD analysis. The vaterite formation indicates the eventual transformation of amorphous conditions into calcite crystals under high CO_2_ pressure and the alkaline conditions of pH (8.5–10.5)^65–67^. The recent studies also have reported that vaterite formation is favored in the presence of amino acids and alcohols in addition to fermented organics (Ramadoss 2019; Ramadoss Ravi 2019). The presence of carbohydrates (C–H stretching) at 1583 cm^-1^ and unsaturated fatty acid peaks at 3394 and 3392 cm^−1^ being observed in all the modified mixes indicates the role of polysaccharides unsaturated fatty acids chains with the lime particle to transform the amorphous hydrated phases to crystalline calcite crystals. The minor stretching of hydroxyl ions at 3340–3494 cm^−1^ indicates ethanol, isopropanol, and calcium hydroxide observed in organically modified samples. It is observed that the reference putty (R.M.) sample has revealed well-developed calcite peaks compared to organic incorporated lime putty samples. However, the unsaturated fatty acid has created a porous surface to increase the carbonation and polysaccharides (starch, cellulose and glucose), slowing down the setting time and as waterproofing surfaces to resist the dampness from the surfaces.

Nevertheless, when the polymer is added as a dry powder, it has to dissolve first before to be able to adsorb on hydrates. Therefore, the hydrates have more time to grow, and the surface area available for adsorption is higher. It is evident that 5% of fermented organics has performed better and can further visualize the high-intensity calcite peaks with higher curing ages.

### FE-SEM–EDX analysis

**Figure 9:**
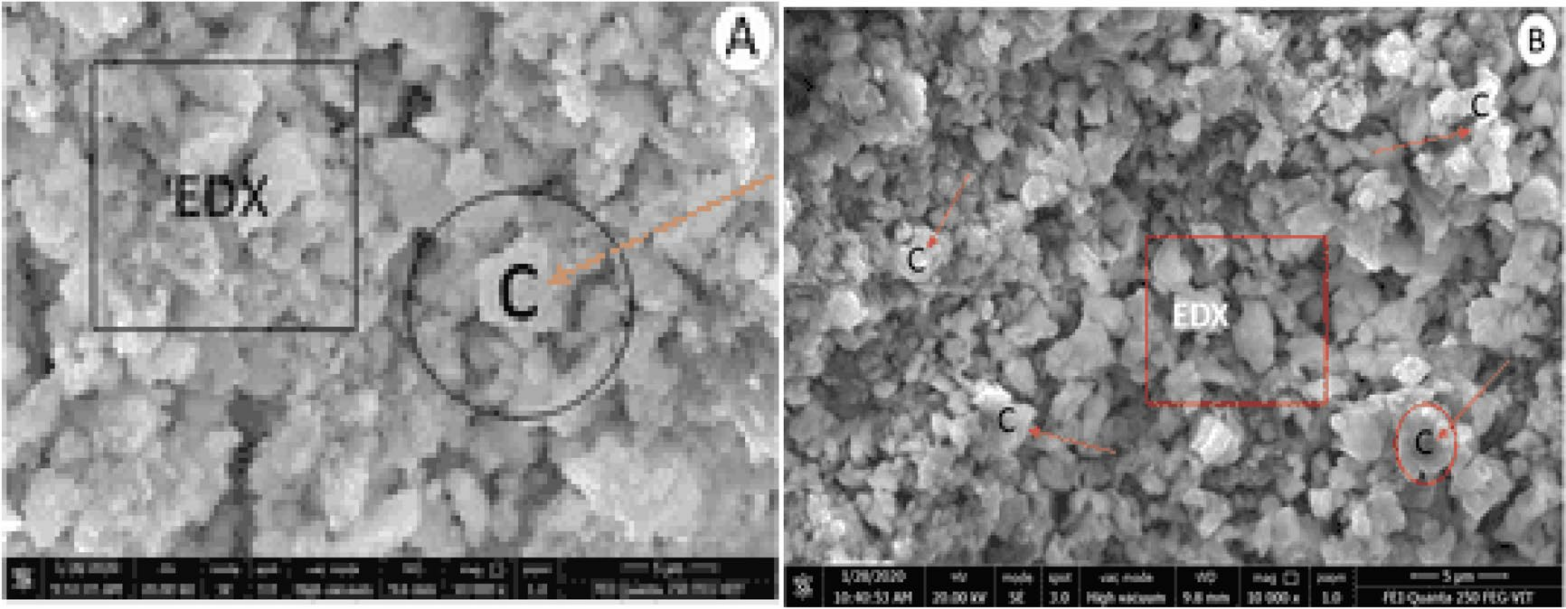
FESEM-EDX Images of modified organic lime paste (**a**). Kadukkai lime paste (**b**). Neelamari lime paste.

**Figure 10:**
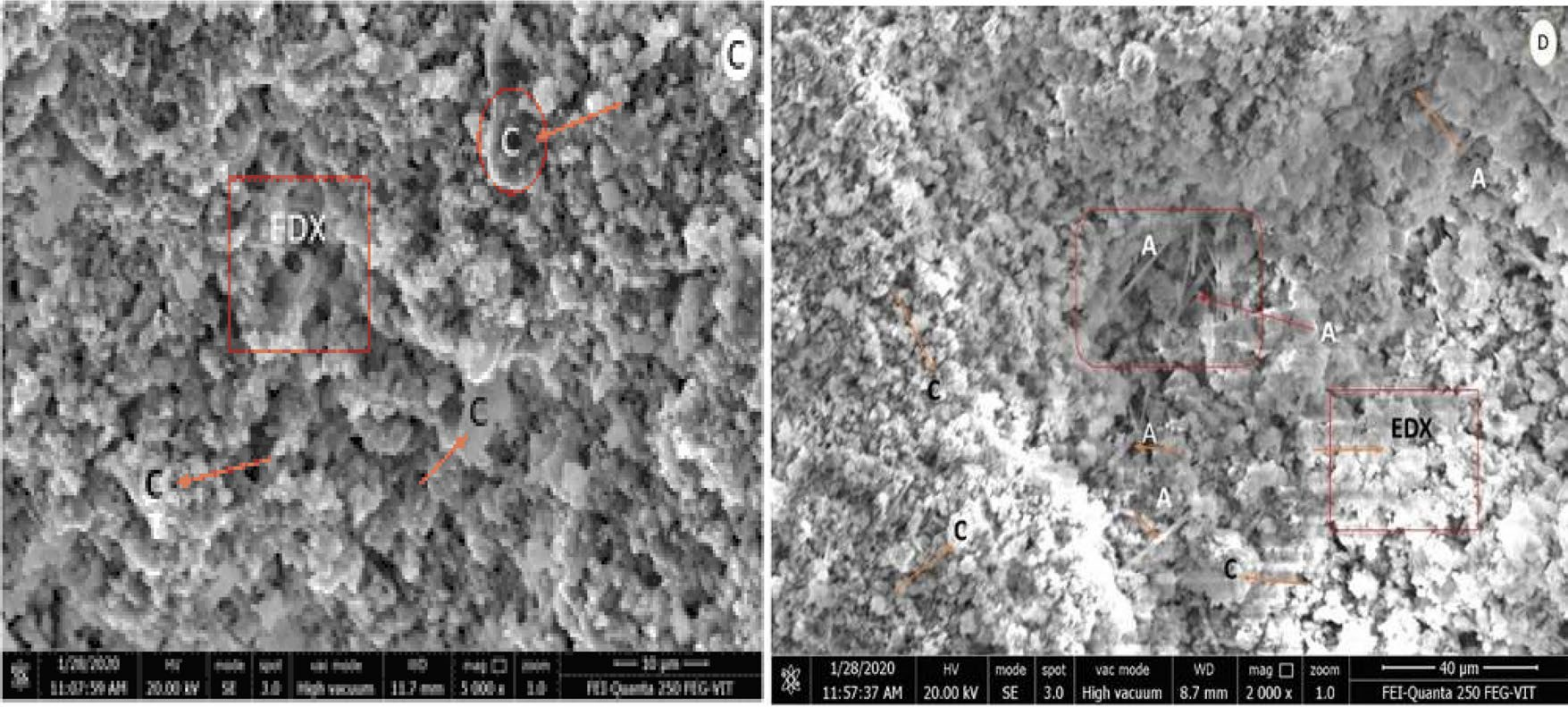
FESEM-EDX Images of modified organic lime paste with calcite and acicular aragonite crystals (**c**). Palm jaggery lime paste (**d**). Aloe vera lime paste.

**Figure 11:**
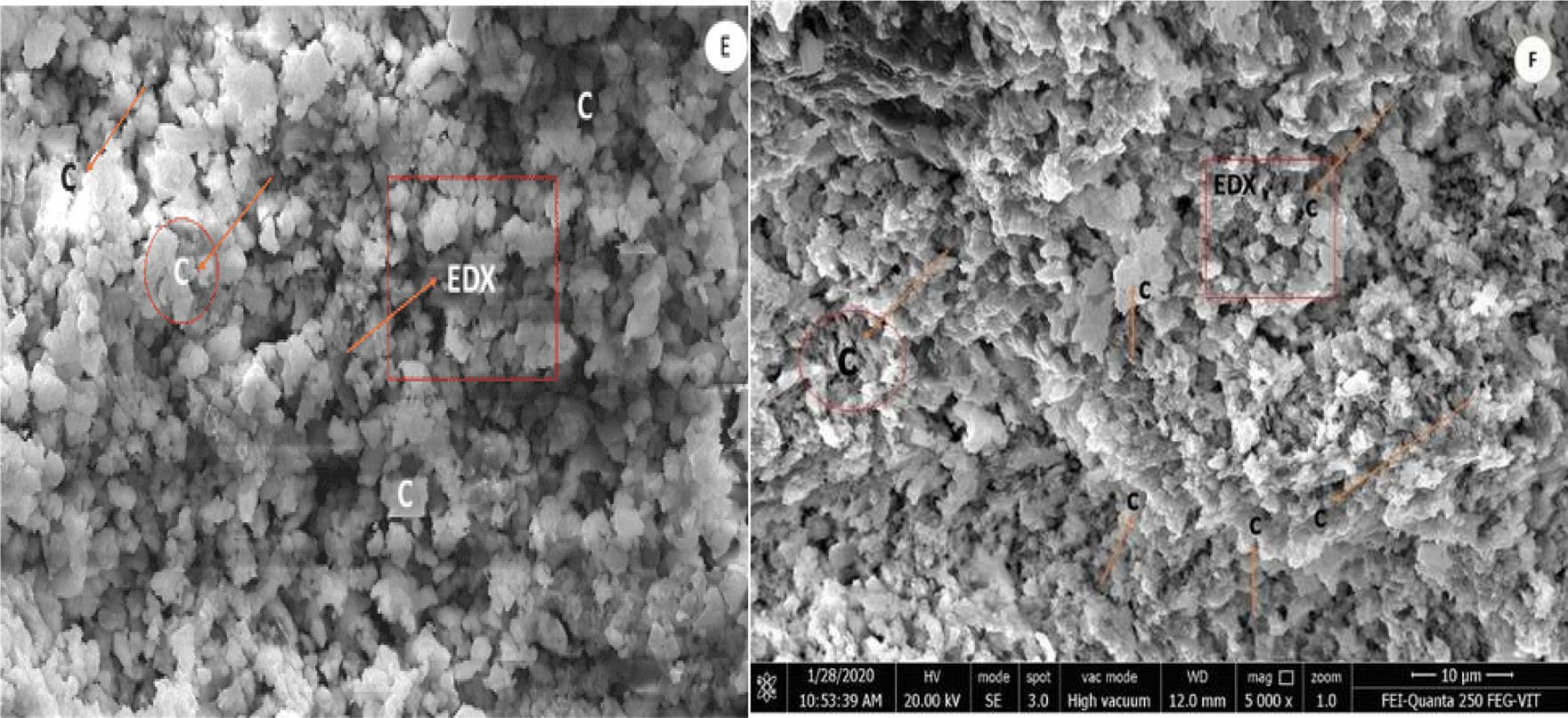
FE-SEM images of modified organic lime paste (**e**).Hibiscus lime paste. (**f**). Reference lime paste. C: Calcite, A; Aragonite.

The morphological information of organic lime paste, provided by the field emission microscopy, helped provide valuable information about the reaction compound, allowing us to observe the nature of formation, sizes, texture and distribution system ranging between 3300 and 10,000 X in Figs. 9, 10, and 11. Reference lime paste revealed the formation of crystalline hydrated phases with E.D.X. spots indicated the more calcite formed compared to organic modified lime paste samples in agreement with XRD and FT-IR analysis. The most striking observation in all the organic lime paste was the amorphous calcite formation and major presence of portlandite peaks in observance to the XRD characterization from Fig. 4. The aloe vera lime paste sample showed the acicular shaped aragonite crystals, resulting in the paste's consistency and enhancing the compressive strength of the paste. Also, this was supported by the E.D.X. spectrum, as shown in Table 13. having the atomic weight percentage of (Ca and Mg) ranging between 35 and 45% along with carbon around 22–25% and other minor fraction of (Si, Fe, Al) around 2–4% respectively in agreement with XRD mineralogical depiction of organic lime paste samples^46^. The amorphous nature hindered the identification of calcite and vaterite crystals in the organic lime paste. It is observed that the surface morphology of all the organic lime paste has revealed well-developed calcite crystals and reduced pore spaces due to the enhanced carbonation in the presence of organic mineral^2,60,67^. The polysaccharides play a prominent role in the lime paste's microstructure and decarbonization properties. In the presence of organic additives, the initial setting has been prolonged. Hence, the cracks are not observed. It indicates that Ca (OH)_2_ and organics like protein and carbohydrates will increase the calcite formation and improved mechanical strength as time progresses^68,69^.

**Table 13:** Elemental analysis (E.D.X.) of modified organic lime paste.

Elemental composition % of organic lime paste and reference mortar using E.D.X						
Elements	Atomic mass weight %					
	KLP	NLP	JLP	HLP	ALP	RM
O	42.82	46.17	28.17	48.1	39.62	26.92
C	9.36	9.97	20.79	23.48	17.14	20.16
Ca	40.79	40.73	43.86	20.54	35.43	46.12
Mg	2.48	1.11	4.23	2.87	3.34	3.65
Fe	0.69	0.93	1.23	1.02	0.68	1.02
Si	2.99	NIL	NIL	1.74	3.46	0.46
Al	0.55	1	0.94	1.66	NIL	0.96
	99.68	99.91	99.22	99.41	99.67	99.29

K.L.P.: kadukkai lime paste, N.L.P.: neelamari lime paste, J.L.P.: Jaggery lime paste, H.L.P.: hibiscus lime paste, A.L.P.: aloe vera lime paste.

## Ancient production technology of Organic lime mortars

Many ancient structures across the countries have documented various indigenously available organic materials during the production technology. To improve strength and durability, they included starches, flours, gums, oils, tree barks, waxes or plant resins, and animal origins such as animal fats or casein, blood, excrements and urine^70–72^. Ancient practices have incorporated the extracted oil and fats from plants and animals to waterproof the surface from the external dampness and capillary rise from the structure's ground level. Vitruvius stated that fig juice, rye dough, hogs, lard, curdled milk, blood and egg whites were employed to toughen and regulate the setting qualities. During the prehistoric periods, the moisture and dampness from the rain harm the structural wall and deteriorate the building materials. To counterbalance the problems, the historic structures were finished with permeable lime mortar to expand, contract, and flex with changes in external temperatures and moisture, hardening the structure on absorbing the CO_2_ from the external environment throughout its service life to increase the serviceability and longevity of the structure.

The addition of chemical additives has made the cement mortar more impervious with the nil absorption rate of CO_2_. The natural organic additives have activated the internal carbonation and help in carbon sequestration. Researchers reported using organic additives has improved the durability and hardness, reduced the drying shrinkage, resistance to traction, acceleration and deceleration of setting time, adhesiveness, and resistance freeze–thaw cycles, increases the plasticity and workability properties^15,16^. Ancient structures exposed to extreme environmental conditions have been subjected to adverse defects and chemical attacks that require repair and replication of similar traditional production technology with indigenous organics to preserve the structure and ancient wisdom.

Fermented organics like kadukkai, neelamari and palm jaggery and aloe vera are observed to be rich in unsaturated fatty acid through the GC–MS analysis. The unsaturated fatty acid compounds from plant origin have liberated maximum CO_2_ emission on anaerobic conditions. The overall release of CO_2_ confirms that the unsaturated fatty acid compounds have better carbonation with the early transformation of Ca (OH)_2_ into carbonate polymorphs to form the very compact structure due to hydrophobic and water repellency factors creating a waterproofing surface. Justnes et al., 2004 reported that fatty acid anions present in oils (unsaturated fatty acid) react with calcium hydroxide as follows Eqs. (5) and (6).

5 $${\mathrm{Ca}}^{2 + } + \, 2{\mathrm{C}}_{XX} {\mathrm{H}}_{XX} {\mathrm{COOH\`a\;Ca }}\left( {{\mathrm{C}}_{XX} {\mathrm{H}}_{XX} {\mathrm{COO}}} \right)_{2} + \, 2{\mathrm{H}}^{ + }$$

6 $${\mathrm{CO}}_{3}^{2 - } + {\mathrm{ Ca}}^{2 + } {\mathrm{\`a\;CaCO}}_{3}$$

Calcium ions from calcium hydroxide react with the carboxyl group (C.O.O.^-^) of the unsaturated fatty acids to form hydrophobic salts of Ca (C_XX_H_XX_COO)_2_; the dissolved CO_2_ salts carbonates the lime putty to improve the carbonate ions^56^ stated that fermented plant extracts containing more monosaturated fatty acids like oleic acid, octadec 9-enoic acid and hexadec 9-enoic acid are more effective producing the hydrophobicity to the mortar^71^ experimented with the effect of oleic acid on the properties of nano-calcium carbonate. They observed a decrease in the precipitates' particle size, which was also confirmed by wang et al. polysaccharides. These were identified in three herbs like kadukkai, palm jaggery and aloe vera in different percentages. The analyzed polysaccharides and proteins like acetamide, lumicolchicine, pentadecane dioic acid, valine (amino acid), cyclodecasiloxane, pentamide and manopyranose are equally classified as water reducers, set retarders and water retention agents that help to retain the moisture content in lime mortar leading to the delay in the setting time^68^. Polysaccharides shall contain both hydrophobic and hydrophilic segments responsible for the delay in Lime since water molecules could be attached to the parts of hydrogen bonds; being unable to escape this moisturizes the mortar mix. The major drawback of polysaccharide in lime mortar is the induced delay of hydration, and its interlocking mechanism of water molecules inside the mortar imparts slow carbonation (Poinot 2015). The addition of sugars up to 2% has water reduce the effect and reduce risk in cracks induced by dry shrinkage. However, excess addition could affect the mortar's compressive strength and water resistance. However, the usage of organics in mortar has significantly improved the properties like durability, longevity and resistance against external environmental conditions.

The ancient sustainable production technology of organic lime mortars and its capacity to sequester carbon dioxide via biomineralization provides valuable inputs to lessen greenhouse gas emissions, causing climate change and variability. Compared to cement production, Lime is substantially adopted in ancient practice because it produced at a lower temperature around 900–1200 °C, resulting in 40% less CO_2_ output and absorption of CO_2_ during its lifetime. The addition of fermented organic extracts introduces internal carbonation within the material's inner core for faster hardening and setting to resist environmental loads acting on the structure throughout its life span. The formation of additional minerals such as metastable forms of calcium carbonate aragonite and vaterite, calcium alkoxides and calcium oxalates with the supply of CO_2_ supports carbon capture and storage. These added organics improve various properties like carbonation, plasticity, water repellency, and the mortar's strength and durability. Bio-Mineral carbonation in organic lime mortars is a safe and novel method for permanent CO_2_ sequestration and offers a potential and stable carbon capture and storage (C.C.S.)^72^.

## Conclusions

From the archival information on the ancient palm-leaf manuscript of Padmanabhapuram palace construction, the ancient production methodology has made us capitalize on the importance of the type of building materials and various organic additives like kadukkai, neelamari, hibiscus, aloe vera and palm jaggery and also its role with the binder materials that has resulted in the long-term durability to the structure.

- The GC–MS analysis revealed various bioactive compounds such as unsaturated fatty acids, polysaccharides, carbohydrates, and proteins. The limit of quantification (LOQ) and detection limit (LOD) value was found statistically significant for sugar and fatty acid components.
- Lime putties of Jaggery, aloevera, and kadukkai mixes have significantly improved the formation of calcite with acicular aragonite crystals in addition to polysaccharides materials such as glycosides, monomers, monosaccharides, mannopyranose, glucopyranose valine (amino acids) and D-glucose. In the presence of polysaccharides, the organic lime paste offered the hydrophobic property on offering better resistance against the absorption of the soluble substances to confront the dampness on the surfaces.
- Hibiscus lime putty rendered the hydrophobic lining in the lime matrix pores in combined action with the effect of fatty acids and sugar monomers, such as hexadecenoic acid n-hexadecenoic acid, 14-octadecadienoate, tetracontane, fumaric acid, 9-octadecene, hentriacontane and rhodopin-glucose.
- The phytochemicals present in the jaggery lime putty have attributed the combined chemical reaction like water repellency, retarding effect, adhesive properties that show the lime particle interaction to arrest the porosity resisting the shrinkage cracks and developing early mechanical strength with complete carbonation on the internal and external surfaces.
- Supply of CO_2_ has initiated the internal carbonation of the lime putty and precipitation of calcite in three different forms aragonite, calcite and vaterite minerals. The XRD depicted the amorphous polymorphs of (calcite and vaterite) peaks, along with traces of CSH products such as hartrurite and larnite at initial stages, indicated the transformation of amorphous calcium silicate hydrate in agreement with FT-IR analysis. FESEM images revealed that the co-existence of amorphous and crystalline calcite enhances the hydration process as more free lime has been available for the reaction. The EDX results depicted the amount of carbon presence up to 24%.
- Scientific research decodes the influence of plant extracts in the lime mortars. It provides valuable input to the State Archeology Department, conservation engineers and architects, NGO’s, and to simulate new organic mortars for repointing, rendering, stucco, and waterproof surfaces to restore and safeguard the ancient structures.

## Supplementary Information

Supplementary Information.

## Abbreviations

XRD: X-ray diffraction
FESEM-EDX: Field emission scanning electron microscopy
FT-IR: Fourier transformation-infrared spectroscopy
GC–MS: Gas chromatography-mass spectroscopy
K.L.P.: Kadukkai lime putty
J.L.P.: Jaggery lime putty
A.L.P.: Aloe vera lime putty
N.L.P.: Neelamari lime putty
H.L.P.: Hibiscus lime putty
L.O.Q.: Limit of Quantification
L.O.D.: Limit of detection
