# Supplementary material for: Preparation and characterization of ancient recipe of organic Lime Putty-Evaluation for its suitability in restoration of Padmanabhapuram Palace, India
Source: Sci Rep. 2021 Jun 24;11:13261. doi: 10.1038/s41598-021-91680-8 (PMC8225893; doi:10.1038/s41598-021-91680-8)
Supplement: Supplementary file 1 — Supplementary Information. [file 41598_2021_91680_MOESM1_ESM.docx]

Preparation and characterization of ancient recipe of organic Lime Putty-Evaluation for its suitability in restoration of Padmanabhapuram Palace, India.

**M. Shivakumar ^1^ Thirumalini Selvaraj ^2*^** **Magesh Peter Dhassaih ^3^**

^1^ Research scholar, Department of Structural and Geotechnical Engineering, Vellore Institute of Technology, Vellore-632014, India.

^2^ Associate professor, Department of Structural and Geotechnical Engineering, Vellore Institute of Technology Vellore-632014, India.

^3^ Senior scientific officer, National Institute of Ocean Technology, Ministry of Earth sciences, Pallikaranai, Chennai-600100, India.

***[*p.thirumalini@yahoo.in*](mailto:p.thirumalini@yahoo.in)


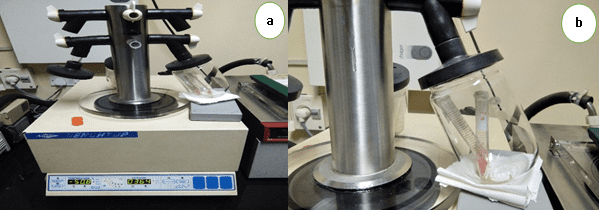


Supplementary Fig 1. (a) Vacuum effect on organic samples (b) Samples converted to pellets after lyophilizing.


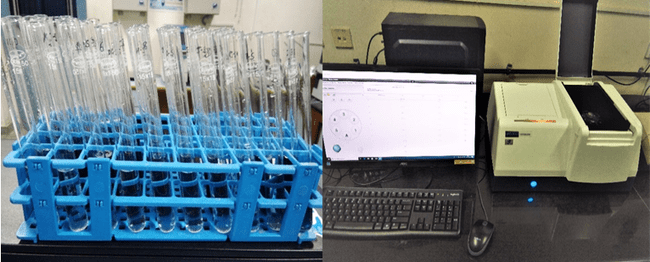


Supplementary Fig 2. Detection of total sugars by UV-Spectrophotometer analysis of organic samples (Dubois method 1956).


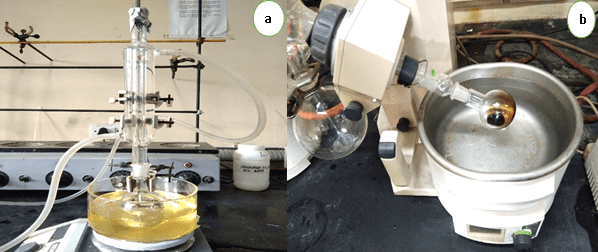


Supplementary Fig 3. (a) Total sugar and fatty acids detection apparatus (Dubois method 1951) (b) Rotary vaporizing the organic samples for GC-MS test.


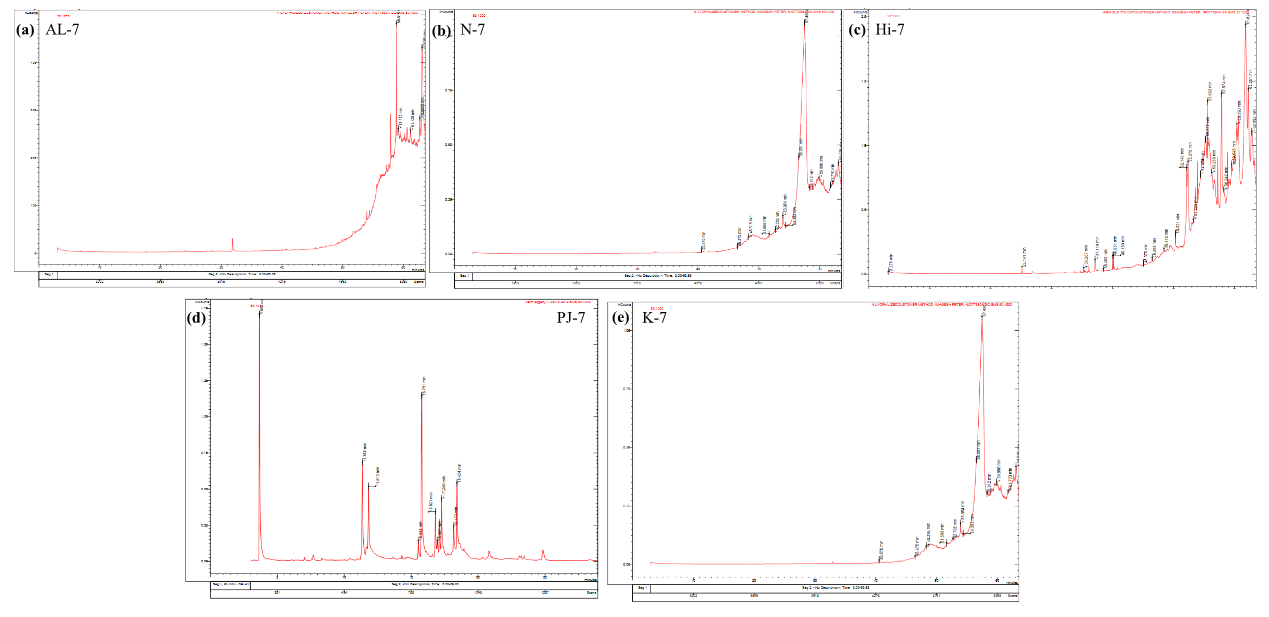


Supplementary Fig 4. (a-e) GCMS simple sugar detection


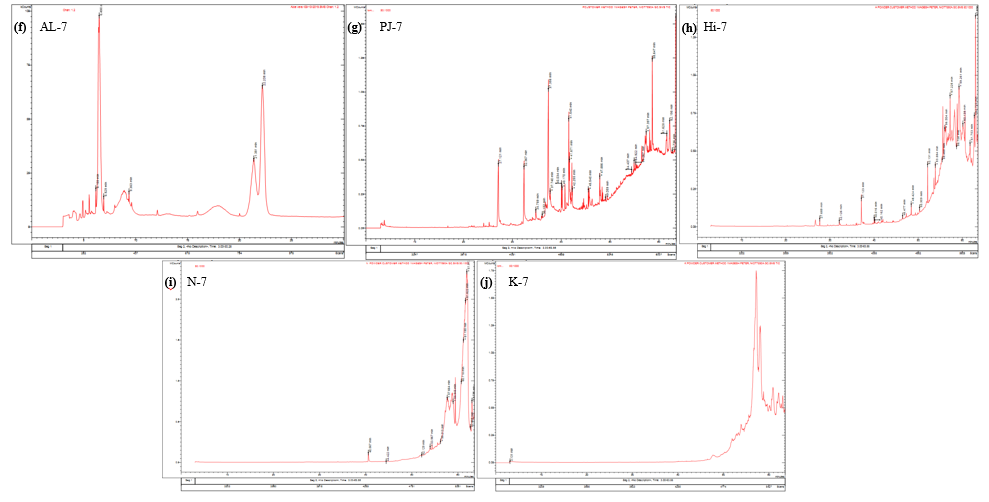


Supplementary Fig 4. (f-j) GC-MS fatty acid detection


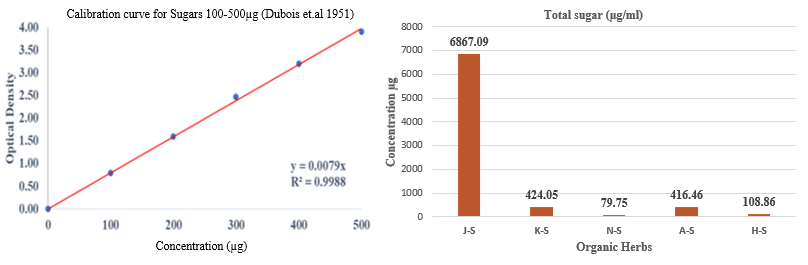


Supplementary Fig 5. Linear regression coefficient of total sugars on absorbance standards (Dubois et al. 1951).


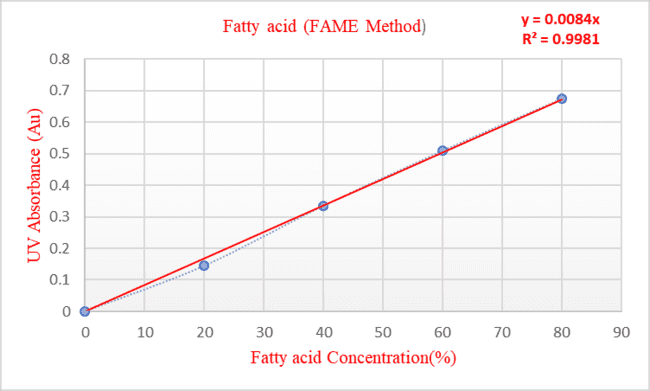


Supplementary Fig 6. Linear regression Coefficient of fatty acids on absorbance standards (Fame method).
